# Targeting ferroptosis: a promising approach for treating lung carcinoma

**DOI:** 10.1038/s41420-025-02308-z

**Published:** 2025-01-29

**Authors:** Ziyang Wu, Yan Zhang, Wendi Zhong, Kunjian Wu, Tian Zhong, Tao Jiang

**Affiliations:** 1 School of Life Sciences, Zhuhai College of Science and Technology, Zhuhai, Guangdong China; 2 Department of Thoracic Surgery, The First Hospital of Jilin University, Changchun, Jilin China; 3 Faculty of Medicine, Macau University of Science and Technology, Taipa, Macao; 4 School of Pharmacy, Faculty of Medicine, Macau University of Science and Technology, Taipa, Macao

**Keywords:** Cell death, Lung cancer

## Abstract

Lung carcinoma incidence and fatality rates remain among the highest on a global scale. The efficacy of targeted therapies and immunotherapies is commonly compromised by the emergence of drug resistance and other factors, resulting in a lack of durable therapeutic benefits. Ferroptosis, a distinct pattern of cell death marked by the buildup of iron-dependent lipid peroxides, has been shown to be a novel and potentially more effective treatment for lung carcinoma. However, the mechanism and regulatory network of ferroptosis are exceptionally complex, and many unanswered questions remain. In addition, research on ferroptosis in the diagnosis and treatment of lung cancer has been growing exponentially. Therefore, it is necessary to provide a thorough summary of the latest advancements in the field of ferroptosis. Here, we comprehensively analyze the mechanisms underlying the preconditions of ferroptosis, the defense system, and the associated molecular networks. The potential strategies of ferroptosis in the treatment of lung carcinoma are also highlighted. Targeting ferroptosis improves tumor cell drug resistance and enhances the effectiveness of targeted drugs and immunotherapies. These findings may shed fresh light on the diagnosis and management of lung carcinoma, as well as the development of drugs related to ferroptosis.

## Facts

- The antitumor mechanism of ferroptosis is achieved via the induction of the abnormal buildup of lipid peroxides.
- Targeting ferroptosis enhances the sensitivity of tumor cells to chemotherapeutic drugs and improves the tumor microenvironment.
- Some drugs used to treat lung carcinoma can trigger ferroptosis.

## Open Questions

- Are there specific and reliable markers of ferroptosis that can be used for the diagnosis and treatment of lung carcinoma?
- How can the sensitivity of cancer cells to ferroptosis be specifically enhanced without affecting normal cell function?
- What is the connection between ferroptosis and other forms of cell death, and can this crosstalk further induce cell death in lung cancer?
- How does ferroptosis affect the tumor immune microenvironment?

## Introduction

Lung carcinoma has the highest incidence and mortality of all malignancies worldwide [1]. Various factors, including long-term smoking, environmental pollution and occupational exposure, are key factors in the increasing incidence of lung cancer [2]. Although traditional chemotherapeutic agents have played a pivotal role in lung cancer treatment, their limitations, including potential toxic effects, imprecise regulation of drug release, relatively low bioavailability, and the rapid development of drug resistance in patients, cannot be overlooked [3]. Furthermore, the heterogeneous and complex nature of lung cancer cells poses significant challenges to monotherapy, driving researchers to delve into the exploration of innovative strategies for combination therapy [4]. In addition, significant breakthroughs in lung cancer treatment have been achieved through immunotherapy and targeted therapy. Notably, immune checkpoint inhibitors (ICIs) have revolutionized the treatment of non-small cell lung cancer (NSCLC), leading to substantial improvements in patient survival outcomes [5]. In the realm of targeted therapy, drugs targeting specific gene mutations such as EGFR (epidermal growth factor receptor) have demonstrated remarkable efficacy in certain patient subpopulations. However, the application of these agents is also accompanied by drug toxicity, the emergence of resistance, and high costs [6]. Therefore, exploring an efficient and safe treatment for lung cancer has become an urgent need for current research.

Ferroptosis is a mode of cell death that relies on iron and is triggered by the buildup of reactive oxygen species (ROS) along with lipid peroxides in cellular membranes [7]. Morphologically, ferroptosis is characterized by disruption of plasma membrane integrity, swelling of both the cytoplasm and organelles, and a moderate degree of chromatin condensation [8]. Ferroptotic cells display ultrastructural alterations in the mitochondria, including diminished size, heightened density of the bilayer membrane, and loss of mitochondrial cristae, whereas the nucleus remains consistently normal in size [9]. These morphological characteristics distinctly differentiate ferroptosis from other modalities of cell death. The mechanism of ferroptosis causing cell death makes it highly promising for tumor treatment. First, cancer cells depend particularly on ferroptotic defense mechanisms to combat oxidative stress caused by elevated levels of ROS [10]. Second, ferroptosis is significantly linked to the treatment resistance observed in lung cancer. For example, erastin promotes ferroptosis, which helps reduce the resistance of NSCLC to radiation treatment [11]. Last, both oncogenic genes and tumor suppressor proteins exhibit intricate interactions with ferroptosis. For instance, p53 increases the vulnerability of tumor cells to ferroptosis by modulating the system Xc^-^ [12]. Conversely, the cellular resistance to ferroptosis is enhanced by increased oncogenic KRAS; however, inhibitors of ferroptosis have demonstrated efficacy in treating cancers harboring KRAS mutations [13]. In conclusion, ferroptosis is strongly correlated with tumor cell growth and invasion, as well as drug resistance. In this paper, we provide a comprehensive overview of the advances in ferroptosis research related to lung cancer, highlighting both the mechanisms that trigger ferroptosis and the strategies for cellular defense against it. In addition, we explored the molecular network of ferroptosis in lung carcinoma, which may be useful for the development of anti-lung carcinoma drugs that target ferroptosis. Strategies aimed at targeting ferroptosis in lung carcinoma were also analyzed to guide future research directions.

## Essential prerequisites for the induction of ferroptosis

### Iron overload

**Fig. 1:**
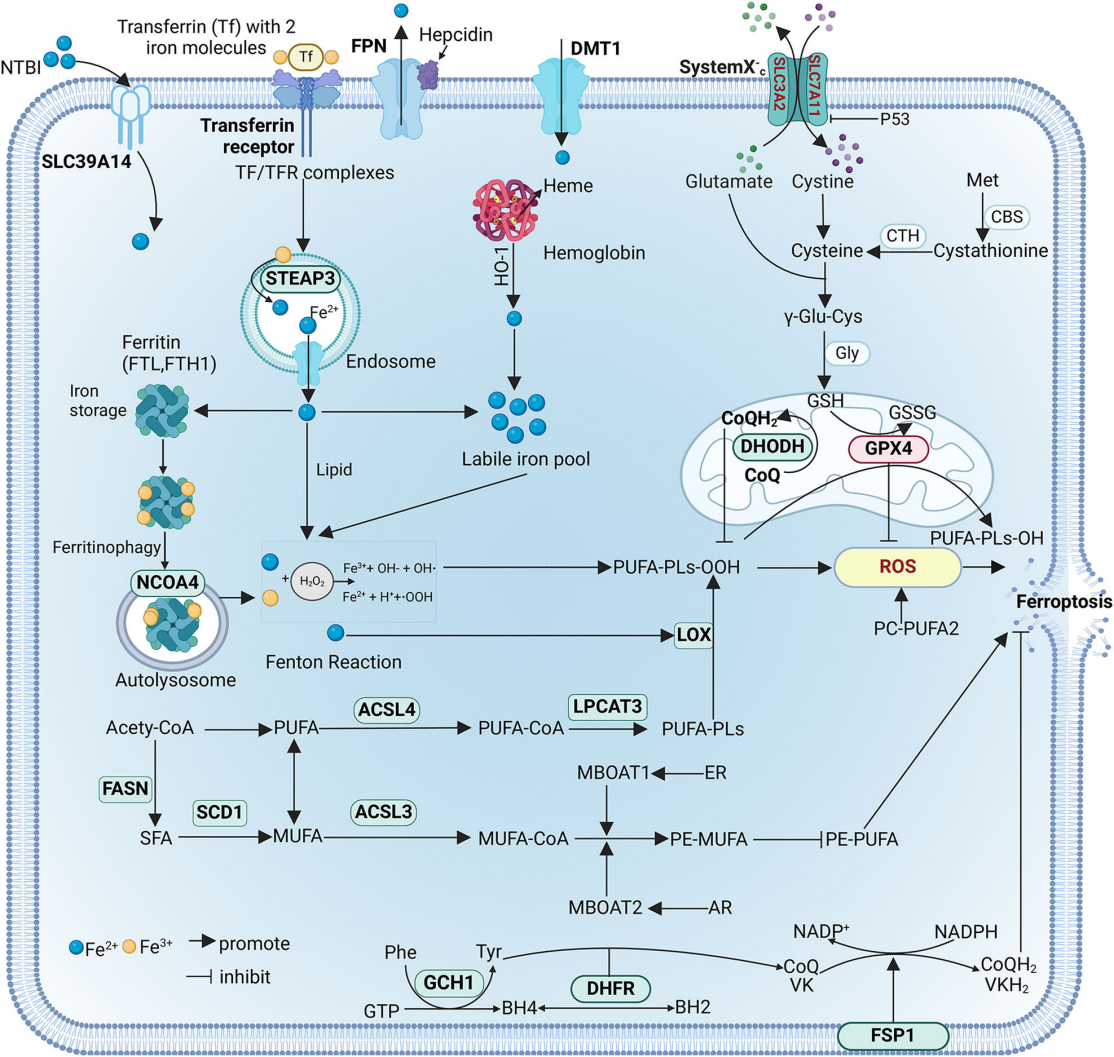
Potential molecular mechanism of ferroptosis. Ferroptosis is mainly induced through iron overload, lipid peroxide accumulation, and redox imbalance. In iron overload, TF, FTH, FPN, and NCOA4 are responsible for regulating the level of Fe^2+^ in cells. In lipid peroxidation, ACSL4 and LPCAT3 can convert free PUFAs into PUFA-PLs, thereby inducing ferroptosis. Additionally, ACSL3 converts MUFA into MUFA-CoA, which may indirectly inhibit the formation of PUFA-PLs. FASN and SCD1 regulate the saturation of fatty acids. In redox regulation, cystine is transported into the cytoplasm through the Xc^-^ system for the synthesis of GSH. GSH assists GPX4 in eliminating excessive lipid peroxides in cells. Additionally, both FSP1 and DHFR exert anti-ferroptosis effects by generating CoQ_10_H_2_.

Iron overload is an indispensable prerequisite for ferroptosis [14]. SLC39A14 is situated on the cell membrane and can allow the cell to take up nontransferrin-bound iron (NTBI) [15]. In addition, transferrin (TF) transports free iron from the blood to target tissues and organs. Iron-loaded TF and transferrin receptor (TFR) bind to form complexes, which are internalized via endocytosis and then reduced from Fe^3+^ to Fe^2+^ by the transmembrane iron-reducing enzyme STEAP3. Divalent metal transporter 1 (DMT1) can transport Fe^2+^ out of the endosome and transport NTBI into the cell [16] (Fig. 1). Excess intracellular iron is stored in the labile iron pool (LIP) to prevent ferroptosis in cells. The intracellular degradation of heme or hemoglobin can increase the level of LIP [17]. However, excessive iron accumulation leads to the generation of radicals through the Fenton reaction, which then interact with polyunsaturated fatty acids (PUFA) and cause ferroptosis [18]. Ferritin is another crucial factor that affects LIP. Ferritin is composed of a heavy chain (FTH) and a light chain (FTL), which can convert Fe^2+^ to Fe^3+^. However, autophagy of ferritin facilitated by nuclear receptor coactivator 4 (NCOA4) triggers ferroptosis by liberating iron into the cytoplasm [19]. Ferroportin (FPN) is an iron-exporter protein that transports excess iron out of cells and is regulated by hepcidin [20]. Collectively, the induction of ferroptosis occurs when the influx of iron surpasses its efflux and storage, leading to the iron overload.

### Lipid peroxide accumulation

Lipid peroxides represent the most immediate factor that initiates cell membrane rupture during ferroptosis. Phospholipids (PLs) on the cell membrane, especially PUFA-PLs, including arachidonic acid-PL and adrenalic acid-PL, are very susceptible to peroxidation and drive ferroptosis [21]. ACSL4 (acyl-CoA synthetase long-chain family member 4) and LPCAT3 (lysophosphatidylcholine acyltransferase 3) are essential for catalyzing the conversion of free PUFAs to PUFA-PLs [22] (Fig. 1). ACSL4 facilitates the esterification of PUFAs with long-chain acyl-coenzyme A, leading to the creation of PUFA-CoAs. LPCAT3 subsequently incorporates these PUFA-CoAs into phospholipids (PLs) through re-esterification, leading to the synthesis of PLs containing PUFAs (PUFA-PLs) [23]. During ferroptosis, Fe^2+^ and lipoxygenases (LOXs) hold a significant role in oxidizing PUFA-PLs, resulting in the subsequent generation of PUFA-PLs-OOH [24, 25]. In addition, a recent study revealed that PC-PUFA2 (phosphatidylcholine with two polyunsaturated fatty acid tails) also has strong potency and specificity in inducing ferroptosis. Its interaction with the mitochondrial electron transport chain elicits the generation of ROS, which subsequently initiates the process of lipid peroxidation [26]. Monounsaturated fatty acids (MUFAs) are also important for modulating lipid peroxidation. ACSL3 catalyzes the transformation of MUFAs to MUFA-CoA, which is less susceptible to peroxidation than PUFA-PLs [7]. In addition, FASN (fatty acid synthase) and SCD1 (stearoyl-coenzyme A desaturase 1) are crucial for modifying the lipid composition of the cell membrane. FASN uses acetyl-CoA as a substrate to synthesize saturated fatty acids (SFAs), whereas SCD1 can convert SFAs to MUFAs, thereby reducing the sensitivity of cells to ferroptosis [27–29]. Additionally, the enzymes MBOAT1 and MBOAT2 play essential roles in regulating membrane lipid composition by enhancing the incorporation of MUFAs into phosphatidylethanolamine (PE). This biochemical modification leads to an increase in MUFA-PE concentrations and a concurrent reduction in PUFA-PE levels, ultimately sensitizing tumor cells to ferroptosis. Notably, the transcription and activity of MBOAT1/2 are subject to modulation by the estrogen receptor (ER) and androgen receptor (AR), which presents a potential avenue for therapeutic intervention in hormone-responsive tumors [30].

### Excessive ROS

Ferroptosis is a process that results from the iron-mediated buildup of lipid peroxides triggered by excessive ROS. Mitochondria play a crucial role in the promotion of ROS production through electron transfer, while NADPH oxidases (NOXs) and xanthine oxidases (XOs) also produce additional ROS [31]. When the rate of ROS generation exceeds the clearance capacity of the body, there is an excessive accumulation of ROS, which react with Fe^2+^ to induce lipid peroxidation, thereby initiating ferroptosis.

## Defense mechanisms against ferroptosis

### System Xc^-^/GSH/GPX4 axis

The system Xc^-^ transporter is composed of SLC7A11 (solute carrier family 7 member 11) and SLC3A2 (solute carrier family 3 member 2) [32, 33] (Fig. 1). It primarily mediates the exchange of cystine and glutamate. Imported cystine is then involved in the synthesis of glutathione (GSH). GPX4 (glutathione peroxidase 4) employs GSH as a cofactor to facilitate the detoxification of lipid peroxides into lipid alcohols, hence playing a pivotal role in preventing ferroptosis [34, 35]. Conversely, the downregulation of GPX4 or GSH results in the inability of cells to scavenge lipid peroxides effectively, thereby inducing ferroptosis [36]. Additionally, under conditions of intracellular system Xc^-^ limitation, cysteine synthesis can occur via the methionine transsulfuration pathway. This metabolic route is facilitated by CBS (cystathionine beta-synthase) and CTH (cystathionine gamma-lyase) [37].

### FSP1

FSP1 (Ferroptosis suppressor protein 1) acts as an NADH-dependent oxidoreductase that reduces ubiquinone (CoQ_10_) to ubiquinol (CoQ_10_H_2_) and vitamin K to vitamin K hydroquinone (VKH_2_) [38, 39]. CoQ_10_H_2_ and VKH_2_, lipophilic antioxidants that scavenge free radicals, can inhibit the buildup of lipid peroxides, thereby preventing the onset of ferroptosis [39]. Additionally, FSP1 is endowed with the capacity to synthesize the intermediate metabolite 6-OH-FAD, which not only functions as a crucial cofactor in enhancing the activity of FSP1 but also directly exhibits antioxidant properties by trapping free radicals. This dual functionality of 6-OH-FAD effectively dampens lipid peroxidation processes, thereby exerting a suppressive effect on ferroptosis [40] (Fig. 1).

### GCH1/BH_4_ axis

GCH1 (GTP cyclohydrolase 1) is responsible for catalyzing the conversion of GTP to BH4 (tetrahydrobiopterin), which is a potent antioxidant capable of effectively neutralizing intracellular free radicals and hindering lipid peroxidation, thereby impeding ferroptosis. Additionally, GCH1 promotes the production of reduced CoQ_10_, enhancing the cellular antioxidant capacity and protecting cell membranes from oxidative damage [41].

### DHODH

Similar to FSP1, DHODH (dihydroorotate dehydrogenase) impedes ferroptosis by converting CoQ_10_ to CoQ_10_H_2_. The difference is that this process occurs in the inner membrane of the mitochondria instead of the cellular membrane [42].

### Lysosomal exocytosis

Lysosomal exocytosis refers to the mechanism through which lysosomes are discharged from within the cellular milieu and transported to the extracellular space. AKT enhances the binding of TRPML1 to ARL8B by phosphorylating TRPML1, thereby triggering lysosome exocytosis. This process reduces the accumulation of Fe²⁺ and subsequently decreases lipid peroxide generation, thus inhibiting ferroptosis [43].

### Other antioxidant systems

In addition to GPX4, VKH_2_, and CoQH_2,_ which act as scavengers for free radicals, ferroptosis can also be mitigated by antioxidants such as α-tocopherol [44], superoxide dismutase (SOD), and catalase (CAT) through efficient neutralization of ROS. Furthermore, the P62/Keap1/Nrf2 axis also suppresses ferroptosis by inhibiting oxidative stress [45].

## Modulation of ferroptosis in lung cancer

### Modulation of iron metabolism in lung cancer

**Fig. 2:**
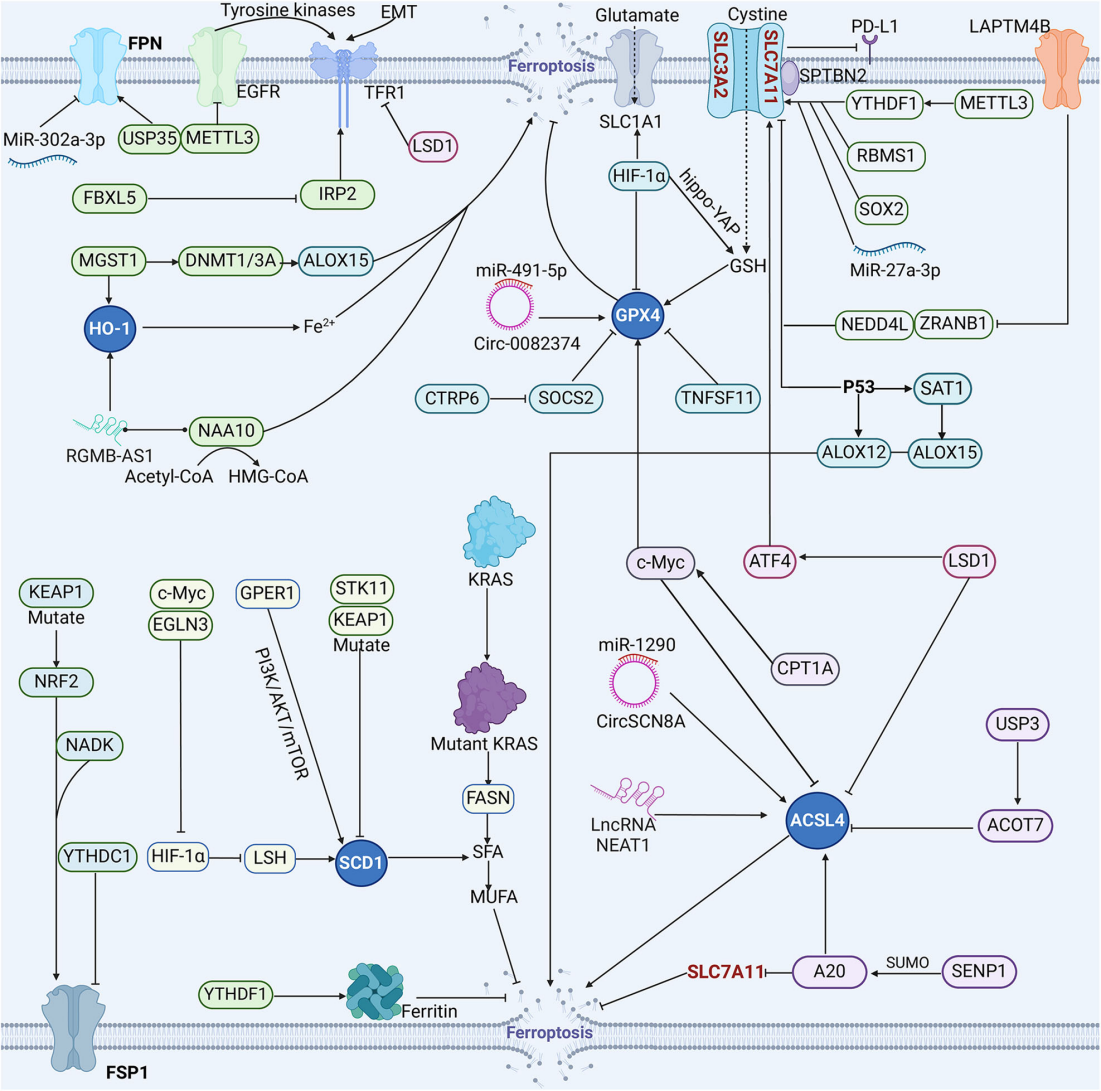
Intricate regulatory network of ferroptosis in lung cancer. HO1, GPX4, SCD1, ACSL4, SLC7A11, and FSP1 are the major regulatory sites. MGST1 and RGMB-AS1 are responsible for regulating the expression of HO1 and inducing ferroptosis. TNFSF11, HIF-1α, CTRP6, and Circ-0082374 regulate GPX4 expression. EGLN3, GPER1, and STK11/KEAP1 complexes regulate the expression of SCD1 and inhibit ferroptosis. USP3, SENP1, CPT1A, LncRNA NEAT1, and CircSCN8A regulate the expression of ACSL4. In addition, FSP1 is regulated by KEAP1, NRF2, NADK, and YTHDC1. LAPTM4B, METTL3, RBMS1, SOX2, and MiR-27a-3p promote SLC7A11 expression, while p53 inhibits SLC7A11 expression.

TFR is a crucial molecule involved in iron absorption at the cellular membrane and is markedly elevated in various malignant tumors [46]. Dowlati et al. confirmed a significant elevation in the TFR in patients with NSCLC relative to those with chronic obstructive pulmonary disease (COPD) [47]. Moreover, elevated TFR expression in lung cancer cells undergoing EMT (epithelial-mesenchymal transition) has been demonstrated to facilitate iron acquisition and confer heightened sensitivity to ferroptosis in these cells [30] (Fig. 2). EGFR is a pivotal oncogenic factor that is frequently mutated in various tumors. Wang et al. reported that EGFR regulates the expression of the TFR through the tyrosine kinase pathway, thereby affecting the iron uptake capacity of lung cancer cells [48]. These studies present novel curative strategies for lung cancer patients who have undergone EMT and who have EGFR mutations. Methyltransferase-like 3 (METTL3) is an RNA methyltransferase involved in the m^6^A modification of RNA. In lung cancer cells, METTL3 can simultaneously regulate the mRNA stability of TFR1 and SLC7A11, which in turn decreases cellular vulnerability to ferroptosis [49, 50]. However, in glioblastoma, METTL3 diminishes ferroptosis sensitivity through stabilizing GPX4 expression [51]. IRP2 (iron regulatory protein-2) is vital for the regulation of iron metabolism. The downregulation of IRP2 in A549 cells significantly reduced the levels of TFR [52]. Therefore, a reduction in IRP2 expression in lung cancer cells may be associated with a poor prognosis [53]. Additionally, Hinokuma et al. revealed that leucine-rich repeat protein 5 (FBXL5) serves as an iron sensor and that its deficiency causes the accumulation of IRP2, thereby leading to increased Fe²⁺ levels in lung cancer cells [54]. The similar regulatory mechanism of IRP2 has also been proven in other types of tumor cells [55].

Ferritin can convert highly reactive iron (Fe^2⁺^) to less reactive iron (Fe³⁺) and store it, thereby leading to a decrease in the sensitivity of lung cancer cells to ferroptosis. Consequently, ferritin may also be an important regulatory central target [56]. YTHDF1 (YTH domain family member 1‌) accelerates ferritin translation in a m^6^A-dependent manner and reduces the susceptibility of lung cancer cells to ferroptosis [57]. In COPD, YTHDF1 facilitates the translation of IREB2 (Iron-responsive element-binding protein 2), consequently inducing ferroptosis [58]. FPN is also crucial in the regulation and preservation of cellular iron homeostasis. The deubiquitinase USP35 is responsible for targeting FPN to ensure its stability. Downregulation of USP35 expression increases the vulnerability of lung cancer cells to ferroptosis and diminishes their resistance to chemotherapeutic drugs, including cisplatin and paclitaxel [59]. Interestingly, in renal clear cell carcinoma, USP35 confers resistance to ferroptosis by catalyzing the deubiquitination of NRF2 [60]. MicroRNA 302a-3p downregulates the protein production of FPN by specifically targeting its 3’ untranslated region (3’ UTR). Consequently, this downregulation results in increased iron accumulation within cells, ultimately inducing ferroptosis in lung cancer cells [61].

Heme oxygenase-1 (HO-1) facilitates the breakdown of heme, leading to the liberation of unbound iron and the onset of ferroptosis. In NSCLC, the lncRNA RGMB-AS1 interacts with HO-1, increasing its stability and potentiating ferroptosis by impeding ubiquitination mediated by the E3 ligase TRC8. Furthermore, by binding to N-α-acetyltransferase 10 (NAA10), RGMB-AS1 also enhances the transformation of acetyl-CoA to HMG-CoA, which results in increased ferroptosis [62]. Interestingly, reduced expression of HO-1 may also increase the likelihood of ferroptosis. Ma et al. discovered that the knockdown of microsomal glutathione S-transferase 1 (MGST1) significantly reduced the protein levels of HO-1 and DNMT1/3 A, leading to decreased DNA methylation in the promoter region of arachidonic acid 15-lipoxygenase (ALOX15). This process triggers ferroptosis in NSCLC cells and enhances their susceptibility to radiation therapy [63].

### Modulation of lipid metabolism in lung cancer

ACSL4 plays a vital role in the production of PUFA-PLs. A tobacco exposure experiment demonstrated that the expression level of ACSL4 in smokers was much greater than that in nonsmokers. This observation suggests that the upregulation of ACSL4 may be a potential contributing factor to lung cancer [64]. LncRNA NEAT1 shows overexpression in multiple tumors, and its expression level is significantly associated with tumor cell proliferation [65]. Wu et al. determined that the NEAT1 negatively regulates ACSL4 expression via a targeted interaction, resulting in decreased sensitivity of NSCLC cells to ferroptosis [66] (Fig. 2). However, in hepatocellular carcinoma, the upregulation of NEAT1 leads to an increased level of myo-inositol oxygenase and enhanced ROS production, consequently increasing the sensitivity to ferroptosis [67]. Similarly, Liu et al. revealed that circSCN8A acts as a competitive endogenous RNA to increase ACSL4 transcription by binding to miR-1290, thereby inducing ferroptosis and effectively inhibiting NSCLC cell proliferation and metastasis [68]. Furthermore, carnitine palmitoyltransferase 1 A (CPT1A), an enzyme that limits the rate of fatty acid oxidation, can inhibit c-Myc ubiquitination. The CPT1A/c-Myc signaling axis reduces lipid peroxide generation in lung cancer cells by suppressing ACSL4 expression and concurrently enhances resistance to ferroptosis through stimulation of the NRF2/GPX4 antioxidant pathway [69]. Acyl-CoA thioesterase 7 (ACOT7) catalyzes the hydrolysis of fatty acyl-CoA [70]. Tao et al. found that ubiquitin-specific protease 3 (USP3) upregulated the expression of the ACOT7 protein through a deubiquitination mechanism, whereas downregulation of USP3 markedly elevated the expression of ACSL4 and promoted ferroptosis in NSCLC cells [71]. Gao et al. found that SENP1 regulates the inflammatory signal A20 through sumoylation to promote ACSL4 expression or inhibit SLC7A11. This regulatory mechanism promotes ferroptosis in lung cancer cells [72]. Finally, inhibiting lysine-specific demethylase 1 (LSD1) not only elevated the expression of TFR and ACSL4 but also reduced the production of activated transcription factor 4 (ATF4), which in turn lowered xCT expression [73].

Fatty acid synthesis enzyme (FASN) and SCD1 are other important regulatory sites in lipid peroxidation [74, 75]. The KRAS protein is a small GTP hydrolysis enzyme (small GTPase) that frequently undergoes mutation in NSCLC. KRAS-mutant lung cancer cells enhance the biosynthesis of saturated and monounsaturated fatty acids, as well as their corresponding phosphatidylcholine, by upregulating FASN, thereby conferring resistance to oxidative damage and ferroptosis. In contrast, the inhibition of FASN leads to a decrease in these phosphatidylcholine levels and triggers ferroptosis [76, 77]. Moreover, Wohlhieter et al. discovered that comutations in STK11 and KEAP1 suppress SCD1 expression, thereby promoting MUFA synthesis and reducing PUFA levels, which ultimately suppresses ferroptosis in lung cancer cells [78]. Moreover, Jiang et al. demonstrated that EGLN3 (egl-9 family hypoxia-inducible factor 3 gene) and c-Myc activate Lyme-specific helicase (LSH) in lung cancer by inhibiting HIF-1α (hypoxia-inducible factor-1α) [79]. However, the elevated levels of LSH stimulate the upregulation of SCD1 expression, concurrently inhibiting lipid peroxidation and iron accumulation. These effects collectively serve to protect cells from undergoing ferroptosis [79, 80]. G protein-coupled estrogen receptor (GPER1) also increases NSCLC resistance to ferroptosis by stimulating the PI3K/AKT/mTOR axis, which upregulates SCD1 expression [81]. Similarly, the mechanism by which SCD1 inhibits ferroptosis has also been validated in various other types of tumor cells [82, 83].

### Modulation of the redox pathway in lung cancer

p53 is an essential tumor suppressor. In lung cancer cells, p53 downregulates SLC7A11 expression to impede cellular cysteine uptake, resulting in diminished GPX4 activity and heightened vulnerability to ferroptosis [84] (Fig. 2). In addition, Chu et al. reported that p53 can facilitate ferroptosis through direct activation of ALOX12 or indirect activation of ALOX15 via the induction of spermidine N1-acetyltransferase 1 (SAT1) in H1299 lung cancer cells [85–87]. In NSCLC, Yan et al. found that lysosome-associated protein transmembrane 4 beta (LAPTM4B) stabilizes SLC7A11 by inhibiting NEDD4L/ZRANB1-mediated ubiquitination of SLC7A11 [88]. Moreover, RBMS1 (RNA binding motif single-stranded interacting protein 1) expression was significantly elevated in lung cancer and is closely related to tumor metastasis [89]. RBMS1 directly interacts with the translation initiation factor eIF3d of SLC7A11 to increase the translation of SLC7A11, thereby inhibiting ferroptosis [90]. Furthermore, SLC7A11 expression is also directly stimulated by the stem cell transcription factor SOX2 in lung cancer stem-like cells (CSLCs). However, mutations within the SOX2 gene lead to a downregulation of SLC7A11 expression, hence increasing the vulnerability of lung cancer cells to ferroptosis [91]. Deng et al. found that the interaction between spectrin beta nonerythrocyte 2 (SPTBN2) and SLC7A11 in NSCLC is facilitated by its CH domain, which in turn connects it to the motor protein Arp1. This connection is pivotal for maintaining the stability of SLC7A11 [92]. Furthermore, SLC7A11 upregulation in A549 cells diminishes PD-L1 expression and suppresses ferroptosis, suggesting an association between ferroptosis and immunotherapeutic outcomes [93]. Finally, the binding of miR-27a-3p to the 3ʹUTR of SLC7A11 results in a reduction in its transcription, thereby increasing erastin-induced ferroptosis [94].

In A549 cells, Zheng et al. demonstrated that the downregulation of HIF-1α led to a decline in GSH and GPX4 levels via the activation of the Hippo–YAP signaling pathway [95]. In addition, HIF-1α can also activate SLC1A1 expression and promote glutamate uptake, which in turn activates cystine uptake and ultimately inhibits ferroptosis [96]. C1q/tumor necrosis factor-related protein-6 (CTRP6) is a pivotal gene linked to the prognosis of lung cancer patients. Cai et al. reported that CTRP6 promoted the ubiquitination of suppressor of cytokine signaling (SOCS2), which enhances the downstream xCT/GPX4 axis and thus inhibits ferroptosis [97]. Tumor necrosis factor superfamily 11 (TNFSF11) is also significantly expressed in LUAD and is inversely correlated with GPX4 expression. Li et al. demonstrated that an elevated level of TNFSF11 diminishes GPX4 expression and increases sensitivity to ferroptosis inducers [98]. Finally, circ_0082374 enhances GPX4 levels through competitive interactions with miR-491-5p. Therefore, circ_0082374 depletion reduces NSCLC proliferation and the EMT process by decreasing GPX4 expression [99].

FSP1 is also a common regulatory site in oxidative-reduction systems. The tumor suppressor YTH domain containing 1 (YTHDC1) participates in the m^6^A methylation of FSP1, leading to reduced stability of FSP1 and increased vulnerability of lung cancer cells to ferroptosis [100]. Furthermore, FSP1 is a key effector in the KEAP1-NRF2 axis. Typically, KEAP1 represses NRF2 transcription; however, in KEAP1-mutated lung cancer cells, the upregulation of NRF2 results in increased FSP1 expression, thereby facilitating ferroptosis induction [101]. Recently, Meng et al. found that nicotinamide adenine dinucleotide kinase (NADK) is specifically expressed in LUAD. The downregulation of NADK can modulate the levels of NADPH and FSP1, thereby enhancing the sensitivity of LUAD cells to erastin/RSL3-induced ferroptosis [102].

## Strategies involving ferroptosis in the treatment of lung cancer

### Targeting ferroptosis susceptibility

Numerous natural compounds have the potential to increase the susceptibility of lung cancer cells to ferroptosis. 6-Gingerol is a naturally occurring phenolic compound extracted from *Zingiber officinale Roscoe* [103]. Tsai et al. revealed that 6-gingerol enhances the susceptibility of lung cancer cells to ferroptosis and suppresses their proliferation by promoting Beclin 1-dependent autophagy [104] (Table 1). Similarly, curcumin, a phenolic antioxidant, not only promotes ferroptosis in A549 CD133^+^ cells by inhibiting the GSH-GPX4 and FSP1-CoQ_10_-NADH pathways [105] but also increases therapeutic efficacy against NSCLC through the activation of autophagy-dependent ferroptosis in A549 cells [106]. Furthermore, corosolic acid (CA) [107], timosaponin AIII (Tim-AIII) [108], and bufotalin (BT) [109] have been shown to increase the susceptibility of lung cancer cells to ferroptosis by specifically targeting GPX4. However, simply increasing the vulnerability of tumor cells to ferroptosis is insufficient for permanently killing them, and combining these natural products with other therapies may synergistically enhance their efficacy.

**Table 1:** Mechanisms by which therapeutic factors induce ferroptosis in lung cancer cells.

Therapeutic agents	Type of study	Targets	Mechanisms	References
6-gingerol	In vivo: BALB/cNude mice	NCOA4	Suppressed lung cancer cell growth by inhibiting the expression of USP14 and regulating autophagy-dependent ferroptosis.	[104]
	In vitro: A549 cells			
Curcumin	In vivo: NOD/SCID mice, Female C57BL/6 mice	GPX4, FSP1	Inhibited the GSH-GPX4 and FSP1-CoQ_10_-NADH pathways.	[105, 106]
	In vitro: A549 H1299 cells			
CA	In vivo: BALB/c nude mice	GPX4	Decreased GPX4 expression by inhibiting the expression of YAP and GSS	[107]
	In vitro: H1299, A549, PC9, NCI-H520, NCI-H460 cells			
Tim-AIII	In vivo: C57BL/6 J mice, BALB/c-nu/nu nude mice	GPX4	Promoted GPX4 degradation by targeting HSP90.	[108]
	In vitro: H1299, A549, SPC-A1 cells			
BT	In vivo: BALB/c nude mice	GPX4	Inhibited the expression of GPX4 and increased intracellular iron ion levels.	[109]
	In vitro: A549 cells			

### Increased sensitivity to targeted therapy and chemotherapy

The ferroptosis mechanism provides a new approach to overcoming resistance in chemotherapy and targeted therapy. Inducing ferroptosis in lung cancer cells can enhance the sensitivity of drug-resistant cells to therapeutic interventions, thereby improving the effectiveness of anti-tumor medications [110, 111]. Extensive studies have shown that various chemotherapeutic agents, including cisplatin, doxorubicin, and temozolomide, are capable of triggering ferroptosis in tumor cells [112–114]. However, ferroptosis inducers can also strengthen the effects of these chemotherapeutic agents, resulting in accelerated death of drug-resistant tumor cells [115]. Natural compounds such as ent-kaurane diterpenoid (compound 23) [116], α-hederin [117], ginkgetin [118], isoorientin (IO) [119], and d-borneol [120] were found to enhance the therapeutic effect of cisplatin by increasing the sensitivity of lung cancer cells to ferroptosis (Table 2). Moreover, the intravenous anesthetic propofol, which acts on the nervous system, has been shown to reduce the resistance of NSCLC cells to cisplatin by upregulating the levels of miR-744-5p and miR-615-3p while downregulating GPX4 expression [121]. The lncRNA ITGB2-AS1 is upregulated in cisplatin-resistant NSCLC. Chen et al. revealed that ITGB2-AS1 inhibits p53-mediated ferroptosis by stimulating the FOSL2/NAMPT axis, which promotes cisplatin resistance in NSCLC cells. Therefore, targeting ITGB2-AS1 to elicit ferroptosis represents a promising strategy for the treatment of cisplatin-resistant NSCLC [122]. In lung cancer cells resistant to paclitaxel, Zhang et al. discovered that tripartite motif 6 (TRIM6) inhibits glutamine metabolism to prevent ferroptosis. GPNA (L-γ-glutamyl-p-nitroanilide) and BPTES (bis-2-(5-phenylacetamido-1,3,4-thiadiazol-2-yl) ethyl sulfide) effectively suppress TRIM6 function, therefore augmenting the sensitivity of lung cancer cells to paclitaxel [123–125].

**Table 2:** Mechanisms for overcoming resistance to chemotherapeutic agents and targeted drugs by inducing ferroptosis in lung cancer.

Drugs	Ferroptosis inducers	Targets	Mechanisms	References
Cisplatin	Compound 23	GSH	Inhibited GSH expression by inhibiting peroxiredoxin I/II (Prdx I/II).	[116]
	α-hederin	SLC7A11 and GPX4	Activated the DDIT3/ATF3 pathway by inhibiting the expression of SLC7A11 and GPX4, thereby promoting nuclear translocation of EGR1.	[117]
	propofol	GPX4	Inhibited GPX4 levels by upregulating miR-744-5p/miR615-3p expression.	[121]
	Ginkgetin	SLC7A11	Downregulated SLC7A11 expression by inhibiting Nrf2/HO-1 signaling pathway.	[118]
	Isoorientin	Nrf2 and GPX4	Downregulated the expression of GPX4 by suppressing KEAP1/Nrf2 signaling pathway.	[119]
	d-borneol	PCBP2 and PRNP	Increased intracellular iron ion transport by upregulating PRNP and downregulating PCBP2.	[120]
Paclitaxel	GPNA and BPTES	SLC1A5	Inhibited TRIM6 function by suppressing SLC1A5 expression and glutamine uptake,	[123–125]
Erlotinib	β-Elemene	GPX4	Inhibited the expression of GPX4 by upregulating lncRNA H19 and promoting the ubiquitination of GPX4	[128, 129]
Osimertinib	RSL3	GPX4	Induced lipid peroxidation and ROS by binding to GPX4	[131]
Gefitinib	CA9 Inhibitors	TF	Promoted iron uptake and increased intracellular iron levels by inhibiting TF endocytosis	[132]
Sorafenib	JB3	GPX4	Inhibited the expression of GPX4, increased Fe^2+^ accumulation, and promoted lipid peroxidation	[134]

Similar to chemotherapy drugs, targeted drugs also face the challenge of drug resistance. Targeted drugs, including gefitinib, erlotinib and osimertinib, can effectively inhibit the proliferation of lung cancer cells by specifically interfering with the activity of EGFR [126]. However, the nature of cancer is highly intricate and variable, with diverse biomarkers and molecular characteristics even among patients diagnosed with the same type of lung cancer [127]. This heterogeneity implies that not all patients benefit from targeted drugs tailored to specific biomarkers. Moreover, targeted EGFR therapy eventually becomes ineffective in almost all patients due to the emergence of secondary EGFR mutations. β-*Elemene* is a sesquiterpenoid compound derived from *Curcuma aromatica Salisb*. In EGFR wild-type NSCLC, β-elemene can induce ferroptosis by activating TFEB (transcription factor EB) to promote the ubiquitination of GPX4 [128]. However, in EGFR-TKI (tyrosine kinase inhibitor)-tolerant NSCLC, combination therapy with β-elemene and erlotinib *overcomes* resistance by promoting ferroptosis [129] (Table 2). Moreover, certain cancer cells enter a state of quiescent growth during the treatment process, known as drug-tolerant persister (DTP), which allows them to evade the apoptosis induced by chemotherapy and targeted therapy [130]. Konishi et al. discovered that RSL3, *a* GPX4 inhibitor, enhances the susceptibility of DTP to ferroptosis in osimertinib-tolerant PC9 cells [131]. Furthermore, Zhang et al. observed that the upregulation of carbonic anhydrase IX (CA9) has been linked to the development of resistance to gefitinib in lung cells. CA9 *inhibitors increase* iron uptake and increase the susceptibility of lung cancer cells to gefitinib [132]. Sorafenib also has the potential to trigger ferroptosis through downregulating the expression of the STAT3/MCL1 complex [133]. Hence, targeting STAT3/MCL1 may augment the therapeutic outcome of sorafenib in managing NSCLC. Kim et al. developed a sorafenib derivative called JB3, which increases the susceptibility of sorafenib-resistant cells to ferroptosis and has good oral bioavailability [134]. Collectively, the strategy of targeting ferroptosis combined with chemotherapy or targeted therapy has shown a new therapeutic prospect, which may provide more effective and safer treatment alternatives for individuals with lung cancer.

### Enhanced the efficacy of immunotherapy

ICIs have demonstrated considerable promise and efficacy as therapeutic interventions for patients with advanced lung cancer [135]. The main challenge in immunotherapy lies in converting “cold” tumors, which are resistant to the immune response, into “hot” tumors, which are more likely to respond to ICIs [136]. However, the occurrence of ferroptosis has the potential to generate more “hot” tumors by modifying the tumor microenvironment (TME) and influencing the infiltration of tumor-infiltrating lymphocytes (TILs) [137]. Hsieh et al. synthesized zero-valent iron nanoparticles (ZVI-NPs) that downregulated NRF2 expression through regulating the AMPK/mTOR axis, inducing ferroptosis in lung cancer cells. ZVI-NPs also modulated macrophage polarization, augmented the cytotoxic potential of CD8^+^ T cells, and reduced the prevalence of regulatory T cells (Tregs), thereby potentiating the antitumor immune response [138] (Table 3). Similarly, Zhang et al. developed Fe_3_O_4_-based nanoparticles loaded with GOx and the immune-activating peptide tuftsin. GOx can utilize glucose in tumor cells to produce H_2_O_2_, thereby promoting the release of drugs and ferroptosis. Tuftsin can effectively reverse the immunosuppressive microenvironment and recruit effector T cells to lung cancer tissues [139]. Furthermore, several proven ferroptosis inhibitors can also alter the TME. For instance, statins reduce the expression of PD-L1 [140]. Cisplatin induces the polarization of N1 neutrophils, resulting in increased “hot” tumors and consequently enhancing the therapeutic efficacy of ICIs [141]. Dihydroartemisinin (DHA) boosts the immunogenicity of lung cancer cells through triggering macrophages and stimulating CD4^+^ and CD8^+^ T cells [142].

**Table 3:** Mechanisms for enhancing the efficacy of immunotherapy through the induction of ferroptosis in lung cancer.

Therapeutic agents	Ferroptosis targets	Cell lines	Mechanisms	References
ZVI-NPs	NRF2	H1299, H460, A549, MRC-5, IMR-90	Regulated the polarization of macrophages, enhanced the cytolytic activity of CD8^+^ T cells, and reduced the proportion of Tregs	[138]
Fe_3_O_4_-based nanoparticle	GPX4	A549	Regulated the immunosuppressive microenvironment, and promoted the recruitment of effector T cells.	[139]
Statins	GPX4	A549, H1299	Regulated the inflamed TME	[140]
Cisplatin	Lipid peroxidation	LL/2, LLC1	Regulated the polarization of N1 neutrophils	[141]
Dihydroartemisinin	Lipid peroxidation	LL/2, LLC1	Activated macrophages, CD4^+^, and CD8^+^ T cells	[142]

**Fig. 3:**
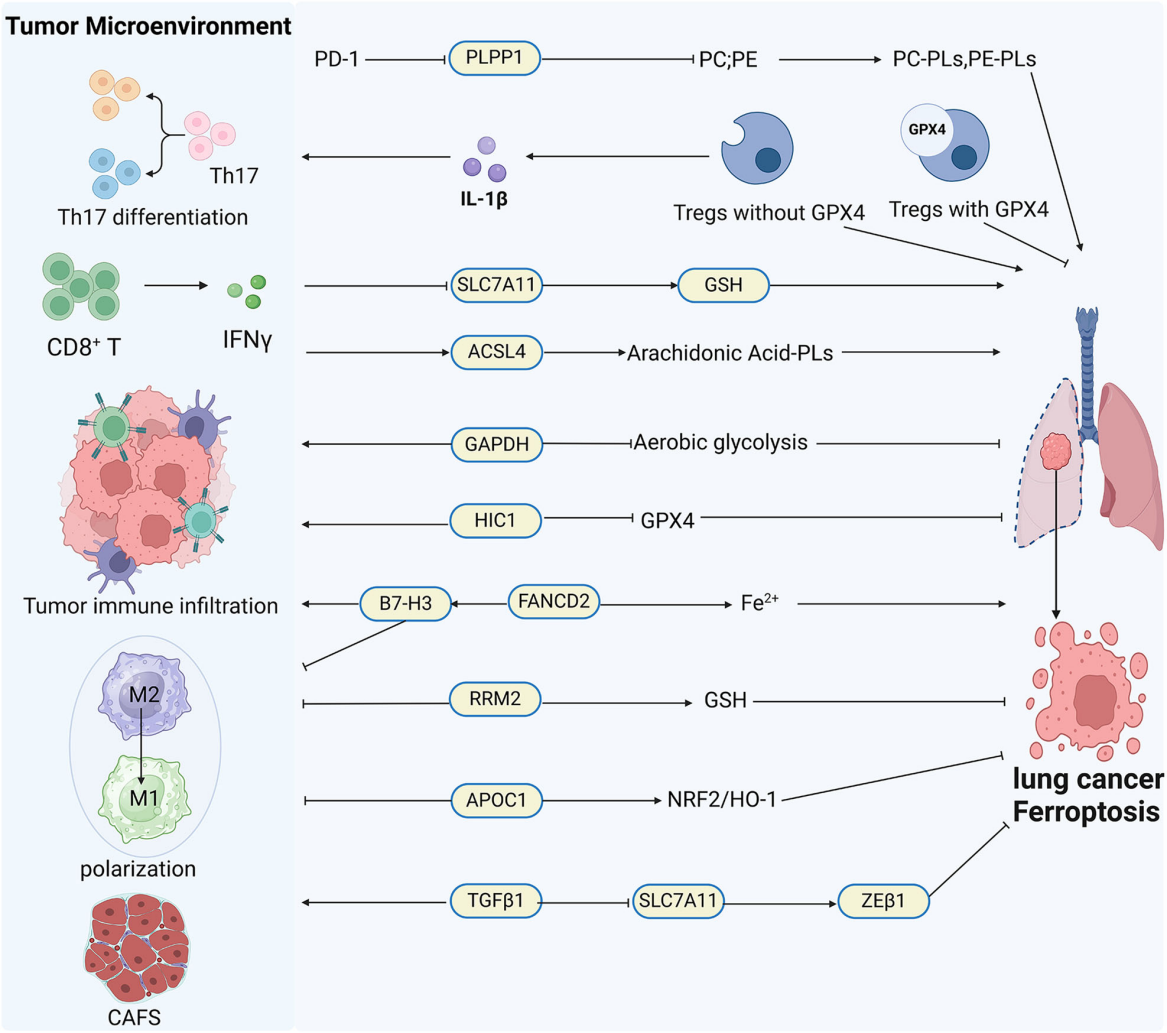
Interplay between ferroptosis and tumor microenvironment in lung cancer. GPX4 promotes Th17 differentiation and activation. IFNγ regulates ferroptosis by inhibiting the expression of SLC7A11 and upregulating the expression of ACSL4. FANCD2, RRM2, HIC1, APOC1, and PLPP1 are potential ferroptosis markers in lung cancer and can enhance immune cell infiltration and macrophage polarization.

The complex relationship between ferroptosis and the TME remains incompletely understood. Xu and colleagues revealed the protective function of GPX4 in Tregs against ferroptosis. Downregulation of GPX4 in Tregs increased the secretion of interleukin-1β (IL-1β), thereby facilitating the differentiation and activation of T helper 17 (Th17) cells [143] (Fig. 3). Moreover, the secretion of IFNγ by CD8^+^ T cells can effectively coordinate the suppression of SLC7A11 expression, thereby activating the ferroptosis pathway, which enhances the antitumor immunotherapeutic effect of anti-PD-L1 antibodies [144]. Additionally, IFN-γ also upregulates ACSL4 and influences the lipid profiles of tumor cells, promoting the synthesis of arachidonic acid-PLs [145]. FANCD2 is a ferroptosis regulator that causes iron accumulation and lipid peroxidation [146]. Ye et al. discovered that FANCD2 is upregulated in LUAD and positively correlates with the immune checkpoint protein B7-H3, which enhances immune cell infiltration and macrophage polarization [147, 148]. Riboiboside-diphosphate reductase subunit M2 (RRM2) reduces cellular sensitivity to ferroptosis by upregulating GSH expression. RRM2 also facilitates immune escape through the promotion of macrophage infiltration and polarization in lung cancer cells [149]. In addition, glyceraldehyde-3-phosphate dehydrogenase (GAPDH) expression is elevated in LUAD patients. Ouyang et al. found that targeting GAPDH can interfere with energy metabolism and suppress aerobic glycolysis within the TME. This strategy increases tumor cell susceptibility to ferroptosis, thereby optimizing the TME and leading to increased immune cell infiltration and improved immune checkpoint activity [150, 151]. HIC1 (hypermethylated in cancer 1), which is implicated in ferroptosis, is significantly lower in lung cancer tissues than in normal counterparts. Further studies revealed that HIC1 induces ferroptosis by repressing GPX4, and its expression is correlated with the infiltration of T cells, B cells, macrophages, and mast cells [152]. TGFβ1, a common component of the TME, has a multifaceted relationship with ferroptosis. It facilitates tumor cell susceptibility to ferroptosis through the suppression of SLC7A11 gene transcription and the activation of ZEB1 activity. However, ferroptotic tumor cells may also reduce the secretion of TGFβ1, which is detrimental to the recruitment of immunosuppressive cancer-associated fibroblasts (CAFs) into the TME [153]. In addition, the combination of a TGF-β inhibitor and an anti-PD-1 antibody can collaboratively establish an immunogenic microenvironment and enhance the Fenton reaction, thereby inducing ferroptosis in tumor cells [154]. In NSCLC tissues, there is an elevation in both the mRNA and protein levels of apolipoprotein C1 (APOC1). Specifically, APOC1 mitigates iron-mediated cytotoxicity by upregulating the NRF2/HO-1 axis. Moreover, APOC1 influences the TME by impeding the polarization of M2 to M1 macrophages. This evidence implies that APOC1 may function as a novel therapeutic target for NSCLC [155]. Additionally, a reduction in phospholipid phosphatase 1 (PLPP1) expression in CD8^+^ T cells from lung cancer tissues is associated with significantly diminished levels of phosphatidylcholine (PC) and phosphatidylethanolamine (PE) compared with their counterparts in peripheral CD8^+^ T cells. The mechanism involves PD-1-mediated induction of GATA1 binding to the Plpp1 promoter in CD8^+^ T cells, resulting in the suppression of Plpp1. Concurrently, this alteration in phospholipid metabolism increases the susceptibility of lung cancer cells to ferroptosis [156].

## Perspectives and conclusions

Despite the remarkable progress in ferroptosis-related research, several challenges remain to be addressed for effectively implementing targeted ferroptosis in lung cancer diagnosis and treatment. First, the intricate nature of ferroptosis regulatory networks, which encompass numerous genes and signaling pathways, poses challenges in uncovering the mechanism of ferroptosis in lung cancer and developing subsequent targeted treatment strategies. The crosstalk between ferroptosis and other modality of cell death also needs further investigation. Second, targeting ferroptosis in lung cancer cells while safeguarding normal cells represents a pressing technical challenge that necessitates urgent resolution. This challenge underscores the importance of investigating specific ferroptosis markers in lung cancer cells [157]. The toxicity and side effects of ferroptosis inducers, such as systemic iron overload and organ damage arising from lipid peroxidation, necessitate rigorous monitoring and management during the therapeutic process [158]. Third, the translation of ferroptosis research achievements into clinical therapeutic modalities necessitates overcoming formidable barriers related to drug delivery, dose optimization, and efficacy evaluation. Finally, given the heterogeneity in tumor biological characteristics and ferroptosis-related pathway states among distinct lung cancer patients, personalized strategies may be needed for ferroptosis-based treatments. Concurrently, the combined application of ferroptosis inducers with other therapeutic modalities poses a critical challenge in optimizing combination regimens to maximize therapeutic outcomes.

In conclusion, this study provides novel insight into the molecular processes involved in ferroptosis, as well as the regulatory network and therapeutic strategies for ferroptosis in lung carcinoma. Circumventing the cellular defenses against ferroptosis in lung cancer cells while maintaining normal physiological functions is crucial for the effective treatment of lung cancer. Strategies that target ferroptosis are anticipated to increase the efficacy of immunotherapy, overcome chemotherapeutic resistance, and increase sensitivity to targeted therapy, suggesting novel treatment options for patients with lung cancer.
